# European Multicenter Study on Coronary Artery Bypass Grafting (E-CABG registry): Study Protocol for a Prospective Clinical Registry and Proposal of Classification of Postoperative Complications

**DOI:** 10.1186/s13019-015-0292-z

**Published:** 2015-06-30

**Authors:** Fausto Biancari, Vito G Ruggieri, Andrea Perrotti, Peter Svenarud, Magnus Dalén, Francesco Onorati, Giuseppe Faggian, Giuseppe Santarpino, Daniele Maselli, Carmelo Dominici, Saverio Nardella, Francesco Musumeci, Riccardo Gherli, Giovanni Mariscalco, Nicola Masala, Antonino S. Rubino, Carmelo Mignosa, Marisa De Feo, Alessandro Della Corte, Ciro Bancone, Sidney Chocron, Giuseppe Gatti, Tiziano Gherli, Eeva-Maija Kinnunen, Tatu Juvonen

**Affiliations:** 1 Department of Surgery, Oulu University Hospital, Oulu, Finland; 2 Division of Cardiothoracic and Vascular Surgery, Pontchaillou University Hospital, Rennes, France; 3 Department of Thoracic and Cardio-Vascular Surgery, University Hospital Jean Minjoz, Besançon, France; 4 Department of Molecular Medicine and Surgery, Department of Cardiothoracic Surgery and Anesthesiology, Karolinska Institutet, Karolinska University Hospital, Stockholm, Sweden; 5 Division of Cardiovascular Surgery, Verona University Hospital, Verona, Italy; 6 Department of Cardiac Surgery, Klinikum Nürnberg, Paracelsus Medical University, Nuremberg, Germany; 7 Department of Cardiac Surgery, St. Anna Hospital, Catanzaro, Italy; 8 Unit of Cardiac Surgery, Department of Cardiosciences, Hospital S. Camillo-Forlanini, Rome, Italy; 9 Department of Cardiac Surgery, Leicester University Hospital, Leicester, UK; 10 Cardiac Surgery Unit, Ferrarotto Hospital, University of Catania, Catania, Italy; 11 Division of Cardiac Surgery, Department of Cardiothoracic Sciences, Second University of Naples, Naples, Italy; 12 Division of Cardiac Surgery, Ospedali Riuniti, Trieste, Italy; 13 Division of Cardiac Surgery, University of Parma, Parma, Italy

**Keywords:** Coronary artery bypass grafting, CABG, complications, E-CABG

## Abstract

**Background:**

Clinical evidence in coronary surgery is usually derived from retrospective, single institutional series. This may introduce significant biases in the analysis of critical issues in the treatment of these patients. In order to avoid such methodological limitations, we planned a European multicenter, prospective study on coronary artery bypass grafting, the E-CABG registry.

**Design:**

The E-CABG registry is a multicenter study and its data are prospectively collected from 13 centers of cardiac surgery in university and community hospitals located in six European countries (England, Italy, Finland, France, Germany, Sweden). Data on major and minor immediate postoperative adverse events will be collected. Data on late all-cause mortality, stroke, myocardial infarction and repeat revascularization will be collected during a 10-year follow-up period. These investigators provided a score from 0 to 10 for any major postoperative adverse events and their rounded medians were used to stratify the severity of these complications in four grades. The sum of these scores for each complication/intervention occurring after coronary artery bypass grafting will be used as an additive score for further stratification of the prognostic importance of these events.

**Discussion:**

The E-CABG registry is expected to provide valuable data for identification of risk factors and treatment strategies associated with suboptimal outcome. These information may improve the safety and durability of coronary artery bypass grafting. The proposed classification of postoperative complications may become a valuable research tool to stratify the impact of such complications on the outcome of these patients and evaluate the burden of resources needed for their treatment.

**Clinical Trials number:**

NCT02319083

## Background

Myocardial revascularization is the most common cardiac surgical procedure. Although the efficacy and long-term durability of coronary artery bypass grafting (CABG) over percutaneous coronary intervention (PCI) has been largely proved in a number of subsets of patients [1, 2], PCI along with its related technological advances has slowly become the most common invasive treatment method for coronary artery disease. The minimally invasive nature and the low risk of immediate adverse events are strong arguments in favor of PCI. However, the current durability of PCI is most likely inferior to that of CABG, particularly in patients with multivessel disease [1, 2], diabetes [2, 3] and renal failure [4].

Contrary to PCI, advancements in coronary surgery during the last decade have been slow and limited to improvements in perfusion and anesthesiological methods. Furthermore, clinical evidence in CABG is usually derived from retrospective and single institutional series and this may introduce significant biases in the analysis of critical issues in the treatment of these patients. However, since CABG is a major surgical procedure associated with postoperative mortality and significant morbidity, there is a need of improvements in the treatment of patients undergoing coronary surgery in order to reduce the risk of early and late adverse events. Prospective analyses of the impact of baseline risk factors, operative techniques, perioperative treatment methods and secondary prevention strategies on the outcome of patients undergoing surgical revascularization of the myocardium are expected to provide valuable and reliable information to improve the safety and durability of CABG.

### Rationale of the study

Improvements in the surgical treatment of cardiac diseases are possible only when implementation of current methods and development of new methods are based on the solid ground of large and reliable clinical data [5]. Knowledge on these issues can be generated by clinical prospective registries. The rationale of this European multicenter study on patients undergoing isolated CABG (E-CABG) is therefore to collect in a prospective fashion current data on baseline characteristics, operative and anesthesiological methods and postoperative outcome of patients undergoing CABG in several European centers of cardiac surgery.

Prospective clinical registries have a relevant role in clinical research as they differ from randomized clinical trials in many aspects. They require less resources and are not narrowly focused on specific subsets of patients, but rather provide data on general patient populations with limited exclusion criteria. Importantly, clinical registries may provide data on long-term outcome that exceed the study window of a trial [5]. Clinical findings of registries assume even more significance when including study populations from different geographic areas with heterogeneous referral pathways, baseline clinical characteristics and perioperative treatment strategies.

The E-CABG has been planned to provide data with well-defined definition criteria and this paper is a summary of baseline, operative and postoperative variables. In particular, these investigators propose a grading system to stratify the severity of postoperative complications in patients undergoing adult cardiac surgery. This study is registered in Clinicaltrials.gov with the number NCT02319083.

## Methods

### Setting, design, study population, patient recruitment and study period

The E-CABG is an observational registry study and its data are prospectively collected consecutively from 13 centers of cardiac surgery of either university or community hospitals located in six European countries (England, Finland, France, Germany and Italy) (Table 1). Patients aged > 18 years undergoing isolated primary or redo CABG for stable coronary artery disease or acute coronary syndrome are eligible for inclusion in this prospective registry. Patients undergoing any other major cardiac surgery procedure will be excluded from this registry. However, patients who undergo CABG associated with Maze will be included in this registry. Patients are recruited in a consecutive series from each institution and their data collected in a specifically created Access-datasheet. The minimum recruitment period is 12 months (January 1 - December 31, 2015). Patients will be followed up after surgery for the following 10 years.

**Table 1:** Participating centers and steering committee members of the E-CABG multicenter trial

	*Participating centers*	*Steering Committee Member*	*Other Investigators*
1	Department of Surgery, Oulu University Hospital, Oulu, Finland	F. Biancari, *Principal investigator*	T. Juvonen
			E.M. Kinnunen
2	Department of Cardiac Surgery, Verona University Hospital, Verona, Italy	F. Onorati	G. Faggian
3	Division of Cardiac Surgery, Ospedali Riuniti di Trieste, Trieste, Italy	G. Gatti	A. Pappalardo
			L. Maschietto
4	Cardiac Surgery Unit, Ferrarotto Hospital, University of Catania, Catania, Italy	C. Mignosa	A.S. Rubino
5	Department of Cardiac Surgery, Klinikum Nürnberg, Paracelsus Medical University, Nuremberg, Germany	G. Santarpino	T. Fischlein
6	Department of Cardiac Surgery, Leicester University Hospital, Leicester, UK	G. Mariscalco	G. Murphy
			N. Masala
7	Department of Thoracic and Cardio-Vascular Surgery, University Hospital Jean Minjoz, Besançon, France	A. Perrotti	S. Chocron
8	Department of Molecular Medicine and Surgery, Department of Cardiothoracic Surgery and Anesthesiology, Karolinska Institutet, Karolinska University Hospital, Stockholm, Sweden	P. Svenarud	M. Dalén
9	Unit of Cardiac Surgery, Department of Cardiosciences, Hospital S. Camillo-Forlanini, Rome, Italy	F. Musumeci	R. Gherli
10	Division of Cardiothoracic and Vascular Surgery, Pontchaillou University Hospital, Rennes, France	V.G. Ruggieri	H. Corbineau
			J.P. Verhoye
11	Department of Cardiac Surgery, St. Anna Hospital, Catanzaro, Italy	D. Maselli	C. Dominici
			S. Nardella
12	Division of Cardiac Surgery, Department of Cardiothoracic Sciences, Second University of Naples, Naples, Italy	M. De Feo	A. Della Corte
			C. Bancone
13	Division of Cardiac Surgery, University of Parma, Parma, Italy	T. Gherli	F. Nicolini

### Data management and monitoring

Data will be collected in to a dedicated Access datasheet with predefined variables. Data including patients’ codes are stored in institutional network and secured by access code. Each Steering Committee Member is in charge for checking the quality and validity of her/his institution’s dataset. Auditing of the dataset will be performed every six months at institutional level by checking the data of 10 % of patients. Data without any patient identification code will be submitted to the principal investigator for further data checking and merging. The merged and checked dataset will be available to all E-CABG investigators for subanalyses.

### Statistical methods

Continuous variables will be reported as mean and standard deviation or median and interquartile range as needed. Dichotomous and nominal variables will be reported as counts and percentages. Missing data will not be replaced. Univariate analysis will be performed using the Mann–Whitney *U* test, Student’s *t*-test, Kruskall-Wallis test, Wilcoxon test, Fisher exact test, Chi-square test and Kaplan-Meier test. Multivariable analyses will be performed using logistic, classification tree, linear and ordinal regression methods as well as the Cox-proportional hazards method. Significant differences between study groups will be adjusted by using propensity score as covariate or one-to-one propensity score matching. Matching will be performed using a caliper width of 0.2 of the standard deviation of logit of the propensity score. Multiple propensity score adjusted analysis will be performed in case of multiple study groups. A Bayesian hierarchical approach will be used in case of significant between-centers variability.

### Immediate and late outcome end-points

The primary outcome end-points will be defined according to the issue investigated in each study. The main immediate outcome end-points of studies from the E-CABG registry are: 1) In-hospital mortality and 30-day mortality, 2) stroke, 3) prolonged use of inotropes, 4) postoperative need of intra-aortic balloon pump (IABP) or extracorporeal mechanical oxygenation (ECMO), 5) immediate repeat revascularization, 6) wound infection and mediastinitis, 7) blood losses and use of blood products, 8) nadir hematocrit, 9) use of prothrombotics, 10) resternotomy for bleeding, 11) atrial fibrillation, 12) acute kidney injury and need of renal replacement therapy, 13) type V myocardial infarction, 14) pericardial effusion requiring treatment, 15) postoperative use of antibiotics, 16) delirium requiring drug treatment, 17) length of stay in the intensive care unit, and 18) length of in-hospital stay. The late outcome end-points of studies from the E-CABG registry are: 1) all-cause mortality, 2) cardiovascular mortality, 3) stroke, 4) myocardial infarction, 5) repeat revascularization and 6) a combined outcome end-point including any of these late adverse events. These end-points and their definition criteria are described in details in the following paragraphs of this article.

### Dissemination policy

The research findings originating from data of the E-CABG registry will be disseminated in the scientific community by presenting the results of these studies in international congresses and publishing them in peer-review international journals in the fields of cardiac surgery and cardiology.

### Steering Committee

The data collection, analysis and writing process will be monitored by the Steering Committee of the E-CABG study. This Steering Committee is formed by a Principal Investigator and a Representative from each of the participating center. Participating centers and investigators are listed in Table 1. The Members of the Steering Committee will take the responsibility for the progress of data collection and its quality through local audit. The Steering Committee will evaluate any study proposal and accept/reject it by voting after having reviewed the study plan and discussed on its feasibility.

### Authorship and the right to use of the registry data

Investigators will be eligible for authorship only if they contributed substantially to study planning, data collection, data analysis and interpretation, writing and critical revision of the manuscripts. In view of the major efforts that each center will face during data collection, one to three authors per each center will be included as main authors of each study performed using the E-CABG registry data. Members of the Steering Committee will decide about any co-authorship from her/his center. The researcher who plans the sub-study, performs the analysis and writes the article will have full right to be the first author of the study. The principal investigator will finalize the database and will guarantee that each Steering Committee Member will have a copy of the overall database as well as of any database created for sub-analyses.

### Research ethics approval

The study has been approved by the local Institutional Review Board or Hospital Chief according to national guidelines for approval of registry studies. Patients’ informed consent is collected in institutions where it is mandatory.

### Schedule

Collection of baseline, operative and immediate postoperative outcome data has started on January 2015 and will end not before December 2015 with a minimum follow-up of 30-days. Follow-up data will be collected during January of each year for ten years. The first analyses of this dataset are expected on July 2015.

### Baseline characteristics and operative data: definition criteria

#### Units of measurements

Units of measurements are likely to differ between centers. In order to avoid any problem during data merging and analysis, laboratory data will be collected according to the suggested units of measurement.

#### Laboratory parameters

Baseline levels of hemoglobin, hematocrit, creatinine, platelets, blood glucose, HbA1c, C-reactive protein, TT-INR and platelets will be collected. Nadir hemoglobin and hematocrit levels as well as highest level of serum creatinine will be recorded as well.

#### Preoperative heart rate and blood pressure

These parameters are obtained in the operating room before anesthesia induction.

#### Preoperative drug treatment

Data on all antithrombotic drugs administered before surgery will be collected. The date of pause of drug treatment refers to the last day the patient received the drug.

Data on any oral or intravenous antibiotics administered preoperatively without prophylaxis purpose, i.e. for any preoperative infectious condition, will be collected.

#### Preoperative *TIA*

Transient ischemic attack refers to any preoperative focal or global neurological syndrome caused by ischemia or hemorrhage resolving within 24 h.

#### Preoperative stroke

Any preoperative focal or global neurological syndrome caused by ischemia or hemorrhage not resolving within 24 h.

#### Poor mobility

Severe impairment of mobility secondary to musculoskeletal or neurological dysfunction.

#### Extracardiac arteriopathy

One or more of the following: claudication, carotid occlusion or >50 % stenosis, amputation for arterial disease, previous or planned intervention on the abdominal aorta, limb arteries or carotids.

#### Diabetes

Diabetes requiring diet, oral or insulin treatment.

#### Hypertension

Arterial blood pressure > 140/90 mmHg or anti-hypertensive treatment.

#### Renal failure

Renal failure will be classified according to the estimated glomerular filtration rate (eGFR) calculated using the Modification of Diet in Renal Disease Study Group modified formula [6]. eGFR for calculation of the EuroSCORE II [7] will be estimated using the Cockcroft-Gault formula [8] according to the criteria of this risk scoring method. The severity of renal failure will be classified in different stages as listed in Table 2.

**Table 2:** Stages of renal failure

*Stages*	*eGFR level (mL/min/1.73 m* ^*2*^ *)*
1	90 or above
2	89 to 60
3a	59 to 45
3b	44 to 30
4	29 to 15
5	Less than 15 or on dialysis

#### Dialysis

Peritoneal or hemo-dialysis before surgery.

#### Functioning renal transplantation

Previous renal transplantation with preserved renal function.

#### Chronic lung disease

Any long term use of bronchodilators or steroids for lung disease.

#### Atrial fibrillation

Paroxysmal or persistent/permanent atrial fibrillation before surgery.

#### NYHA functional classes

These are defined according to the criteria listed in Table 3 [9].

**Table 3:** New York Heart Association functional classes

*Classes*	*Definition*
I	Cardiac disease, but no symptoms and no limitation in ordinary physical activity, e.g. no shortness of breath when walking, climbing stairs etc.
II	Mild symptoms (mild shortness of breath and/or angina) and slight limitation during ordinary activity.
III	Marked limitation in activity due to symptoms, even during less-than-ordinary activity, e.g. walking short distances (20–100 m). 2Comfortable only at rest
IV	Severe limitations. Experiences symptoms even while *at rest*. Mostly bedbound patients

#### Canadian Cardiovascular Society (CCS) class 4

Inability to perform any activity without angina or angina at rest, i.e., severe limitation.

#### Left ventricular function

Left ventricular ejection fraction recently before CABG (in any case before anesthesia induction).

#### Mitral valve regurgitation

Severity of mitral valve regurgitation before CABG graded in classes from 0 to IV.

#### Systolic pulmonary pressure

Systolic pulmonary pressure measured invasively before anesthesia induction. In case a thermodilution catheter is not inserted, systolic pulmonary pressure will be estimated at echocardiography.

#### Coronary syndrome

Coronary syndrome refers to any of these conditions leading to the present hospital admission for CABG: 1) stable angina, 2) unstable angina requiring or not nitrates infusion, 3) non-ST elevation myocardial infarction, 4) ST-elevation myocardial infarction.

#### Urgency of the procedure

This will be graded according to the criteria listed in Table 4.

**Table 4:** Urgency classes of the procedure and their definition

*Urgency*	*Definition*
Elective	Elective procedure for stable angina pectoris
Urgent	Procedure indicated by medical factors which require the patient to stay in hospital to have operation before discharge
Emergency	Procedure performed before the beginning of the next working day after decision to operate. This is further subdivided in four classes:
Emergency class 1	Persistent angina, ECG changes and/or increasing levels of cardiac enzymes despite best medical treatment (nitrates infusion, etc.). No need of inotropes
Emergency class 2	Hemodynamic instability responsive to inotropes
Emergency class 3	Hemodynamic instability unresponsive to inotropes and/or requiring preoperative insertion of IABP
Emergency class 4	Salvage CABG: patients requiring cardiopulmonary resuscitation (external cardiac massage) en route to the operating theatre or prior to induction of anesthesia.
	This does not include cardiopulmonary resuscitation following induction of anesthesia

#### Ventricular arrhythmia

Ventricular tachycardia, ventricular fibrillation or asystole requiring this hospital admission or during hospital stay before CABG.

#### Out-of-hospital cardiac arrest

Cardiac arrest occurred out of the hospital and requiring the present hospital admission for CABG.

#### Critical preoperative status

Ventricular tachycardia or ventricular fibrillation or aborted sudden death, preoperative cardiac massage, preoperative ventilation before anesthetic room, preoperative inotropes or IABP, preoperative acute renal failure (anuria or oliguria <10 ml/hr).

#### Preoperative inotropic support

Preoperative use of epinephrine, norepinephrine, milrinone, amrinone, dobutamine, dopamine, levophed and/or levosimedan.

#### Preoperative use of IABP

Preoperative insertion of IABP before the start of CABG. This refers to any IABP insertion during coronary angiography, percutaneous coronary artery intervention or in the operating room, but before skin incision.

#### Killip classes

These are defined according to the criteria listed in Table 5 [10].

**Table 5:** Killip’s stages of heart failure

*Stage*	*Definition*
I	No clinical signs of heart failure
II	Rales or crackles in the lungs, an S_3_, and elevated jugular venous pressure
III	Frank acute pulmonary edema.
IV	Cardiogenic shock or hypotension (measured as systolic blood pressure <90 mmHg), and evidence of peripheral vasoconstriction (oliguria, cyanosis or sweating)

#### CSHA Clinical Frailty Scale

Preoperative patient’s frailty is graded according to the CSHA scale as listed in Table 6 [11].

**Table 6:** CSHA Clinical Frailty Scale

*Category*	*Definition*
1	Very fit — robust, active, energetic, well-motivated and fit; these people commonly exercise regularly and are in the most fit group for their age
2	Well — without active disease, but less fit than people in category 1
3	Well, with treated comorbid disease — disease symptoms are well controlled compared with those in category 4
4	Apparently vulnerable — although not frankly dependent, these people commonly complain of being “slowed up” or have disease symptoms
5	Mildly frail — with limited dependence on others for instrumental activities of daily living
6	Moderately frail — help is needed with both instrumental and non-instrumental activities of daily living
7	Severely frail — completely dependent on others for the activities of daily living, or terminally ill

#### Grace score 2.0

This risk score is calculated using the on-line calculator available at http://www.gracescore.org/WebSite/WebVersion.aspx and reported as absolute number [12].

#### Syntax score

This angiographic score is calculated using the on-line calculator available at http://www.syntaxscore.com/[13]

#### EuroSCORE II

This risk score is calculated using the on-line calculator available at http://www.euroscore.org/calc.html and reported in percentage. The risk factors included in the EuroSCORE II and collected in the E-CABG registry are defined according to the EuroSCORE II criteria [7].

#### Previous percutaneous coronary artery intervention

This registry included a number of variables to define accurately any details on previous PCI procedures. Data on the type of stent, either drug eluting or bare metal stents, is collected. Titanium stents are herein considered as bare metal stents. Balloon angioplasty refer to a PCI without stenting. Attempt to PCI with failure to pass the wire/stent through the occlusion/stenosis should not be considered as an accomplished PCI procedure. On the contrary, any PCI attempt resulting in coronary artery complication (thrombosis, dissection, perforation, etc.) should be considered as a prior PCI procedure. Details on PCI-related complications are retrieved as well.

#### Indication for CABG after PCI

The indication for CABG after any PCI procedure is defined as 1) stent thrombosis, 2) in-stent restenosis and/or 3) progression of disease.

#### CABG after PCI

Planned CABG after PCI is defined as a staged procedure in patients whose culprit lesions are treated first by PCI and in whom coronary surgery is planned to achieve complete myocardial revascularization immediately or shortly after PCI. Unplanned CABG refers to any surgical revascularization after PCI which was intended to provide definitive invasive treatment for coronary artery disease.

#### Number of diseased vessels

This refers to the number of vessels (one to three) with a stenosis of at least 50 % in the main artery (the left anterior descending artery, the circumflex artery and the right coronary artery) or their major branches.

#### Left main stenosis

This is defined as any stenosis ≥50 % of the left main trunk.

#### Equivalent to left main stenosis

This is defined as stenoses ≥70 % in the proximal segment of both the left anterior descending artery and the circumflex artery.

#### Antibiotic prophylaxis

Data on type and dose of antibiotics administered prophylactically immediately or during surgery are collected.

#### Off-pump surgeon

This refers to surgeons who have operated in the preceding year at least on 20 patients using the off-pump technique.

#### Revascularization technique

Methods of revascularization are reported in Table 7.

**Table 7:** Revascularization techniques

*Technique*	*Definition*
On-pump	Procedure carried out with the use of cardiopulmonary bypass and cardiac arrest
Off-pump	Procedure carried out without the use of cardiopulmonary bypass and cardiac arrest
Heart beating using cardiopulmonary bypass (HB-CPB)	Procedure carried out on beating heart with the use of cardiopulmonary bypass (without cardiac arrest)
Conversion to HB-CPB	Any conversion from off-pump to HB-CPB
Conversion to on-pump	Any conversion from off-pump to conventional surgery with the use of cardiopulmonary bypass and cardiac arrest

#### Epiaortic ultrasound

This refers to epiaortic ultrasound performed to investigate the status of the ascending aorta.

#### Diseased ascending aorta

This is defined as any sign of atherosclerosis in the ascending aorta at palpation or epiaortic ultrasound.

#### Porcelain aorta

This is defined as an extensive circumferential calcification of the ascending aorta.

#### Other intraoperative data

Details of the type of grafts used, number of distal anastomoses, the type of cardioplegia and its temperature as well as the duration of aortic cross-clamping, cardiopulmonary bypass and operation are collected.

#### Drug treatment at discharge

Data on treatment with aspirin, clopidogrel, ticagrelor, warfarin/Coumadin, low-molecular weight heparin, statins and/or ACE-inhibitors are collected.

### Postoperative adverse events: definition criteria

#### Prolonged use of inotropics (>12 h)

This refers to the use of use of epinephrine, norepinephrine, milrinone, amrinone, dobutamine, dopamine, levophed and/or levosimedan for > 12 h after surgery.

#### IABP

This refers to postoperative insertion of an intra-aortic balloon pump device.

#### ECMO

This refers to intra- or postoperative insertion of an extracorporeal mechanical oxygenation device.

#### Resternotomy for bleeding

This refers to any reoperation for hemostasis/removal of hematoma in presence of excessive bleeding with or without hemodynamic problem.

#### Resternotomy for hemodynamic problems

This refers to any reoperation for hemodynamic problems. This can be associated also with excessive bleeding: in such a case, both categories (Resternotomy for bleeding and Resternotomy for hemodynamic problems) are marked.

#### Sternal and leg wound infections

Wound complications are graded according to the Centers for Disease Control and Prevention definitions of surgical site infections [14]. Any sternal or leg wound infection occurring within three months after surgery will be considered as postoperative wound infections.

#### Postoperative atrial fibrillation

It refers to any event of atrial fibrillation/flutter occurred after CABG and detected at ECG, requiring or not pharmacological/electrical cardioversion. Postoperative atrial fibrillation is defined paroxysmal if it is not detected at patient’s discharge. Otherwise atrial fibrillation is defined as permanent.

#### Postoperative stroke

This complication is defined as any focal or global neurological syndrome occurring during the in-hospital stay caused by ischemia and/or hemorrhage not resolving within 24 h. The diagnosis and nature of stroke is made on the basis of findings at computed tomography and/or magnetic resonance imaging of the brain and confirmed by a neurologist. When neurological signs and symptoms disappear before discharge, stroke is defined temporary, otherwise it is defined as permanent.

#### Type 5 myocardial infarction

This adverse event is defined according to the recent definition criteria by Moussa et al. [15] as summarized in Table 8.

**Table 8:** Definition criteria of type V myocardial infarction

*Baseline condition*	*Definition*
1. In patients with normal baseline CK-MB or cTn (I or T)	The peak CK-MB measured within 48 h of the procedure rises to ≥10 × the local laboratory upper limit of normal (ULN), or to ≥5 × ULN with new pathologic Q-waves in ≥2 contiguous leads or new persistent LBBB, *OR* in the absence of CK-MB measurements and a normal baseline cTn, a cTn (I or T) level measured within 48 h of the procedure rises to ≥70 × the local laboratory ULN, or ≥35 × ULN with new pathologic Q-waves in ≥2 contiguous leads or new persistent LBBB.
2. In patients with elevated baseline CK-MB (or cTn) in whom the biomarker levels are stable or falling	The CK-MB (or cTn) rises by an absolute increment equal to those levels recommended above from the most recent pre-procedure level.
3. In patients with elevated CK-MB (or cTn) in whom the biomarker levels have not been shown to be stable or falling	The CK-MB (or cTn) rises by an absolute increment equal to those levels recommended above plus new ST-segment elevation or depression plus signs consistent with a clinically relevant MI, such as new onset or worsening heart failure or sustained hypotension.

#### Delirium requiring drug treatment

Any postoperative neuropsychological syndrome requiring oral or intravenous drug treatment.

#### Blood loss 12 h after surgery

It refers to the amount of postoperative blood losses from mediastinal drainages within 12 h of surgery. Intraoperative blood losses are not taken in to account.

#### Hemoglobin nadir during the operation day

It refers to the lowest level of hemoglobin detected on the day of operation.

#### Hematocrit nadir during the operation day

It refers to the lowest level of hematocrit detected on the day of operation.

#### Highest postoperative creatinine level

It refers to the highest level of serum creatinine detected after surgery during the in-hospital stay.

#### Acute kidney injury

This end-point is defined as an increase in serum creatinine 1.5 -1.9 times the baseline level or serum creatinine increase≥26.5 μmol/l within seven days after surgery. This definition is according to the KIDGO criteria [16], but it considers only the change of postoperative serum levels of creatinine as outcome end-point. Therefore, acute kidney injury will be classified as listed in Table 9.

**Table 9:** Grading of the severity of postoperative acute kidney injury

*Stages*	*Definition*
1	1.5–1.9 times baseline
	OR
	Serum creatinine increase ≥26.5 μmol/l
2	Serum creatinine 2.0–2.9 times baseline
3	Serum creatinine ≥3.0 times baseline
	OR
	Serum creatinine increase ≥353.6 μmol/l
	OR
	Initiation of renal replacement therapy

The need of renal replacement therapy is defined as temporary or permanent (in case the patient dies or is discharged on renal replacement therapy).

#### Red blood cell (RBC) units transfused intraoperatively

It refers to the amount of RBC units transfused intraoperatively (before the time “*at chest closure*”, see below). This does not refer to RBC units transfused before surgery or after the end of surgery.

#### RBC units transfused perioperatively

It refers to the overall amount of RBC units intra- and/or postoperatively transfused since the operation has started and till the day the patient is discharged home or to any other institution, i.e. intraoperative and postoperative. This does not refer to red blood cell transfused before surgery.

#### Use of fresh frozen plasma, solvent/detergent treated, pooled human plasma (Octaplas), platelets, rFVIIa, cryo-precipitate, fibrinogen and prothrombin complex concentrate

This refers to the use of these blood products used at the time of chest closure as well as after the operation until the day of discharge from the hospital. The amount of blood products are reported in homogenous units.

#### Severity of postoperative bleeding

This will be graded according to the Universal Definition of Perioperative Bleeding (UDPB) in adult cardiac surgery [17]. Severe bleeding is defined as UDPB classes 3–4. Because of the complexity of this grading system, the E-CABG study group has developed its own grading system for a simple definition of severity of perioperative bleeding. The E-CABG investigators recognized that the amount of postoperative blood losses 12 h after surgery as defined by the UDPB criteria may not take into account the amount of intraoperative and postoperative bleeding occurring later on after surgery. Therefore the E-CABG investigators proposed a simple classification of perioperative bleeding based on the amount of RBC, platelets, fresh frozen/Octaplas units transfused during the intraoperative and postoperative period until the discharge. The methods adopted to stratify this outcome end-point are described in details in the paragraph “*Classification of the severity of postoperative complications occurring immediately after surgery*”.

#### Early repeated revascularization

This refers to any CABG and/or PCI performed during the same hospital stay for any graft and/or stent-related complication as well as for progression of coronary artery disease.

#### Gastrointestinal complications

This refers to any gastrointestinal complication requiring endoscopy and/or surgical treatment.

#### Length of stay in the intensive care unit

This refers to the number of days the patient was treated in the intensive care unit (ICU) after surgery. Readmission to ICU is also taken in to account in the estimation of ICU stay.

#### Length of in-hospital stay

This refers to the number of days the patient was treated from the day of operation till the day of discharge from the hospital to any rehabilitation unit or home.

#### In-hospital death

It refers to any death occurred in during the same hospital stay in the Institution where CABG was performed.

#### Late death

This refers to death occurring after discharge due to any cause. Specific causes of death are collected to better define whether death is related to any cardiovascular cause.

#### Late stroke

This refers to any focal or global neurological syndrome occurring after discharge and caused by ischemia and/or hemorrhage not resolving within 24 h. The diagnosis and nature of stroke is made on the basis of findings at computed tomography and/or magnetic resonance imaging of the brain and confirmed by a neurologist.

#### Late myocardial infarction

This refers to any myocardial infarction occurring after discharge and requiring medical or revascularization treatment. Since the current definition of type V myocardial infarction requires adequate validation, only myocardial infarction occurring after discharge will be considered as late myocardial infarction.

#### Late repeated revascularization

This refers to any CABG and/or PCI performed after discharge for graft and/or stent-related complication as well as for progression of coronary artery disease.

#### Combined outcome end-point

This refers to any of the main outcome end-point (all-cause mortality, stroke, myocardial infarction and/or repeat revascularization) occurring any time after CABG. Since the current definition of type V myocardial infarction requires adequate validation, only myocardial infarction occurring after discharge will be considered in this end-point.

### Classification of the severity of postoperative complications occurring immediately after surgery

Adult cardiac surgery is associated with significant immediate postoperative mortality. Indeed, early mortality is generally considered a hard outcome end-point, and often the only one, of any analysis evaluating the outcome of cardiac surgery. However, the incidence of non-fatal complications after major surgery is rather high and may have a negative impact on patient’s recovery and the burden of resources necessary to treat such complications. Furthermore, postoperative complications may have a significant impact also on late survival [18–20].

The need of a grading system of postoperative complications has been recognized in surgery as a measure and a means to improve the quality of health care delivery [21]. Therefore valuable ranking systems have been developed for the assessment of quality of surgical treatment as well as a reproducible tool in clinical research [21, 22]. However, the existing ranking systems are more suitable for patients undergoing non-cardiac surgery as they take into account minor complications which are very frequent in patients undergoing adult cardiac experience and which can be considered of minor prognostic importance in this subset of patients. Therefore, any method for grading the severity and prognostic implications of postoperative complications should be specifically tailored for adult cardiac surgery.

There are objective difficulties in ranking the postoperative complications after cardiac surgery as they may be related to a number of preoperative risk factors and perioperative treatment methods and importantly, relevant complications have not been thoroughly investigated in large clinical series. Therefore, there is a lack of data for a proper weighting of the prognostic impact of postoperative complications. Indeed, previous ranking methods used in non-cardiac surgery were not derived from statistical analyses of clinical datasets, but rather were proposed by a few experienced clinicians based on the current knowledge of the clinical impact of any minor and major postoperative complication. A more reliable method to stratify the clinical impact of any complications would be to retrieve the evidence of the negative impact of postoperative complication through meta-analytical methods. However, data for meta-analysis could be limited to a few major complications and may be lacking for many others.

The E-CABG investigators have selected 25 complications or interventions for their treatment (Table 10), which are believed to have a prognostic impact and possibly associated with increased resources for their treatment. These investigators recognized that in many cases, an intervention performed for any complication would provide a better stratification measure than considering the complication itself. Because of this, a number of complications are herein defined as any intervention performed for their specific treatment. These investigators decided to not enlist here some postoperative adverse events because of intrinsic difficulties in defining their true nature (for example: pneumonia, type V myocardial infarction, sepsis), because they are secondary to other more significant adverse events (for example: prolonged tracheal intubation) or any time interval may not capture the real extent of the event (for example: postoperative blood losses). These investigators recognized also the difficulty in identifying a valid definition of postoperative low-cardiac output syndrome. In fact, thermodilution catheter is not routinely used in many centers and the use of a cutoff of postoperative cardiac index to define this event is thus not feasible. Furthermore, postoperative use of inotropes varies significantly between institutions as their use after cardiac surgery is not limited to the treatment of markedly decreased cardiac index. Therefore, in this registry the definition of postoperative severely depressed cardiac function is reserved only to those conditions requiring postoperative insertion of IABP or ECMO.

**Table 10:** Mean, median and interquartile range of score assigned by 24 cardiac surgeons to 25 significant postoperative complications or interventions for their treatment

*Postoperative complication or intervention*	*Mean score*	*Median score*	*Interquartile range*
Transfusion of 1 unit of RBC	0.5	0.5	1.0
Transfusion of 2–4 units of RBC	2.7	2.5	1.0
Transfusion of 5–10 units of RBC	5.2	5.0	2.0
Transfusion of > 10 units of RBC	7.2	7.0	1.0
Reoperation for bleeding	4.6	5.0	1.75
Transfusion of platelets	2.7	2.0	1.75
Transfusion of fresh frozen plasma or Octaplas	2.7	3.0	1.75
Pericardial effusion requiring pericardial fenestration	3.9	4.0	1.0
Postoperative use of antibiotics	1.9	2.0	1.0
Atrial fibrillation	2.3	2.0	2.0
Ventricular fibrillation/asystole	6.8	8.0	3.0
Administration of inotropics > 12 h	3.9	4.0	2.0
Acute kidney injury not requiring renal replacement therapy	3.6	4.0	1.75
Renal failure requiring renal replacement therapy	6.7	7.0	1.75
Deep wound infection of the leg	2.9	3.0	2.0
Deep sternal wound infection	5.0	5.0	2.75
Mediastinitis	6.6	7.0	3.75
Permanent pace-maker implantation	3.3	3.0	2.0
Surgery for gastrointestinal complications	8.1	8.5	1.0
Stroke	7.4	7.0	1.0
Postoperative IABP	5.0	5.0	2.0
Postoperative ECMO	8.6	9.0	1.0
Surgical or percutaneous procedure for technical failure	7.1	7.0	2
Reoperation for hemodynamic instability	7.5	7.5	1
In-hospital death	10.0	10.0	0

Twenty-four E-CABG investigators have stratified the prognostic and severity burden of these 25 complications or interventions for their treatment giving a score from 0 to 10 to each of these adverse events/interventions. A score of 10 was supposed to measure the worst postoperative complication, i.e. patient’s death. Table 10 summarizes the mean, median and interquartile range of these scores.

The medians of these score were used to stratify the prognostic importance of each complication or intervention for its treatment (Table 11). Transfusion of one unit of RBC was considered not a significant event as both its mean and median scores were below 1. These investigators proposed a four grades classification method of postoperative complications in order to classify them according to their increasing severity. The fourth grade includes only the worst postoperative complication, i.e. patient’s death. Multiorgan failure was not herein considered a postoperative adverse events as this grading system includes most of the components of this syndrome. Table 11 summarizes also additive scores which are the rounded medians of Table 10. The sum of these scores for each complication/intervention occurring after CABG will be used as an additive score for further stratification of the prognostic importance of these events in each patient.

**Table 11:** Grading and additive score for postoperative complications or interventions for their treatment

*Grades*	*Postoperative complications or interventions*	*Additive score*
Grade 0		
	None of the below mentioned complications/interventions	0
Grade 1		
	Postoperative use of antibiotics for proven or suspected infection	2
	Atrial fibrillation	2
	Transfusion of platelets	2
	Transfusion of fresh frozen plasma or Octaplas	3
	Transfusion of 2–4 units of RBC	3
	Deep wound infection of the leg	3
	Permanent pace-maker implantation	3
Grade 2		
	Pericardial effusion requiring pericardial fenestration	4
	Acute kidney injury not requiring renal replacement therapy	4
	Transfusion of 5–10 units of RBC	5
	Reoperation for bleeding	5
	Deep sternal wound infection	5
	Postoperative IABP	5
Grade 3		
	Transfusion of > 10 units of RBC	7
	Renal failure requiring renal replacement therapy	7
	Mediastinitis	7
	Stroke	7
	Surgical or percutaneous procedure for technical failure	7
	Reoperation for hemodynamic instability	8
	Ventricular fibrillation/asystole	8
	Surgery for gastrointestinal complications	9
	Postoperative ECMO	9
Grade 4		
	In-hospital death	10

The E-CABG registry has been tailored to collect all the variables for stratification of perioperative bleeding according to the UDPB classification [17]. However, the latter classification is rather complicated and includes a rather large number of differed blood products for treatment and prevention of excessive bleeding. Its use can be limited by the inclusion in this stratification method of the amount of blood losses. In fact, time interval (12 h after surgery) is supposed to not capture the real amount of bleeding occurring after cardiac surgery. Therefore, extrapolating the medians of Table 10, the E-CABG investigators propose a grading of the severity of intra- and postoperative bleeding based on the type and amount of blood products transfused during surgery and the in-hospital stay as well as on the need of reoperation for bleeding (Table 12). The authors propose also an additive score to better grade the need to treat severe intra- and postoperative bleeding.

**Table 12:** Grading and additive score for severity of intra- and postoperative bleeding as measured by the amount of blood products transfused and the need of reoperation for excessive bleeding

*Grades*	*Intervention for treatment of bleeding*	*Additive score*
Grade 0		
	No transfusion of blood products with the exception of 1 unit of RBCs	0
Grade 1		
	Transfusion of platelets	2
	Transfusion of fresh frozen plasma or Octaplas	3
	Transfusion of 2–4 units of RBC	3
Grade 2		
	Transfusion of 5–10 units of RBC	5
	Reoperation for bleeding	5
Grade 3		
	Transfusion of > 10 units of RBC	7

This classification included any transfusion of RBC, platelets, fresh frozen plasma and Octaplas occurred during surgery and after that, during the same in-hospital stay. Preoperative transfusions are not included in this classification

### Practical implications

This multicenter, prospective registry has been planned to investigate a number of controversial issues regarding the prognostic impact of baseline risk factors as well as treatment methods for surgical myocardial revascularization. This registry will provide also data on the role of pre- and postoperative pharmacological treatments on the outcome of patients undergoing CABG. These informations are expected to provide further knowledge on the mechanisms leading to adverse events after CABG and prevent them.
